# The First Garden City

**Published:** 1903-10-24

**Authors:** 


### THE FIRST GARDEN CITY.

Many people were inclined to look upon the objects of the Garden City Association when first formed, some four years ago, as desirable indeed, but entirely visionary and quite outside the range of practical politics. The main idea of the Association is to fight the twin evils of the overcrowding in our large cities, and the slow but sure depopulation of the country, by inducing manufacturers to follow the examples of Mr. Cadbury and Mr. Lever, and move their factories and their workpeople into the country; establishing round about such a nucleus "garden cities"—that is, towns of a limited number of inhabitants, laid out on a definite plan from the first, with ample space for gardens, parks, and places of recreation.

It is good to find that these excellent principles are already bearing practical fruit, and the First Garden City Company, Limited, has been formed, while an estate of about 4,000 acres has been purchased, and the experiment is in a fair way to be almost immediately begun. On Friday, October 9th, a private inspection of the newly-acquired land took place, at the invitation of the directors; and although the weather was not all that could be wished, for once it did not actually rain, and the wide interest taken in the project was shown by the large number of people who availed themselves of the opportunity offered to them. Considerably over 1,000 mustered to luncheon in the marquee put up for the occasion, and if the result is to be in any way commensurate with the enthusiasm with which the scheme is at present supported success should be certain. The land acquired lies near Hitchin, between that town and Baldock, including in its area the villages of Letchworth, Willian, and Norton. It is pretty country, only 35 miles from London, and the Great Northern Railway runs through the estate, which seems likely to lend itself well to the development of the scheme. The railway company is prepared to give facilities for starting the work, and has already erected a temporary station at Letchworth. The greater portion of the estate is to be retained for agricultural purposes, and dividends to shareholders will be limited to a cumulative dividend of 5 per cent. per annum.

Earl Grey, who presided at luncheon, confessed himself to be a complete convert to the scheme of garden cities, though when first he read Mr. Ebenezer Howard's book upon the subject he was sceptical as to its practicability. He explained that it was proposed to limit the population of the city to about 30,000 people, such a number being considered the maximum to inhabit such an area under conditions conducive to healthy, refined, and cultured life. He believed that the money required—£300,000—would soon be forthcoming; he understood that £75,000 had already been subscribed, and he hoped that the many friends of the movement would show their sympathy by each of them subscribing for a few shares in First Garden City, Limited. Other speakers were Lord Peel, Mr. Hudson, Mr. Cadbury, Mr. Ralph Neville, Mr. Lever, and Mr. Howard, the originator of the whole movement. The following resolution, proposed by Lord Peel, was carried with enthusiasm: "That those present at this meeting welcome the experiment being initiated by the First Garden City, Limited, in the building of a modern industrial town, and wish the directors of the company success in their efforts to provide an object lesson in housing and other social reforms."

The speeches showed a very earnest desire to grapple in a practical manner with the great social evils which are daily growing more serious in their effects upon the physical development of the nation. It is evidently becoming widely recognised that to provide other and better accommodation for the inhabitants of our teeming cities is to strike at the root of the housing problem. As Mr. Lever said, to remove children from the slums to the country is to give them that chance in life which belongs of right to every human being, and the Garden City movement is one which not only fights existing evils but provides an alternative. It only remains to echo the hope expressed by more than one speaker that the scheme will be run upon strictly business lines, for only so will ultimate success be secured. Too often plans for the amelioration of social conditions are left to those excellent people who want to hasten the millennium, and will insist upon dealing with things as they would like them to be, rather than as they are. Hence practical people often fight shy of "idealists." A thousand pities surely, for it is practical common sense allied to high ideals which alone can achieve the best results.

For further particulars concerning the Garden City Company application should be made to the secretary, Mr. Thomas Adams, 347–351 Birkbeck Bank Chambers, Holborn, W.C.

